# Kujigamberol Inhibits IFN-γ and IL-2 mRNA Expression and NFATc2 Binding to Their Promoters in Response to a Phorbol Ester and Ionomycin Stimulation

**DOI:** 10.3390/molecules30102214

**Published:** 2025-05-19

**Authors:** Tanpitcha Yodweerapong, Yuto Ueno, Rikako Yamaguchi, Piimwara Yarangsee, Ken-ichi Kimura, Takao Kataoka

**Affiliations:** 1 Department of Applied Biology, Kyoto Institute of Technology, Matsugasaki, Sakyo-ku, Kyoto 606-8585, Japan; 2 The United Graduate School of Agricultural Sciences, Iwate University, 3-18-8 Ueda, Morioka 020-8550, Japan; 3 Center for Social and Biomedical Engineering, Kyoto Institute of Technology, Matsugasaki, Sakyo-ku, Kyoto 606-8585, Japan

**Keywords:** kujigamberol, NFAT, T cells, interferon γ, interleukin-2, interleukin-4, Fas ligand, eomesodermin

## Abstract

Kujigamberol, a dinorlabdane compound isolated from Kuji amber, exerts multiple biological effects, including anti-allergic and anti-inflammatory activities. The present study demonstrated that kujigamberol inhibited cytokine production by T cells. In response to a phorbol 12-myristate 13-acetate (PMA) and ionomycin (IM) stimulation, kujigamberol suppressed interferon-γ (IFN-γ) and interleukin-2 (IL-2) mRNA expression in murine T-cell lymphoma BW5147 cells stably transfected with the T-box transcription factor eomesodermin. IL-4 and Fas ligand mRNA expression was also inhibited by kujigamberol. In the murine cytotoxic T-cell line CTLL-2, kujigamberol more strongly decreased IFN-γ mRNA expression induced by IM alone than that induced by the combination of PMA and IM. A luciferase reporter assay showed that kujigamberol preferentially reduced nuclear factor of activated T cell (NFAT)-dependent transcription in human embryonic kidney 293T cells. Unlike the calcineurin inhibitor FK506, kujigamberol did not markedly affect NFATc2 protein levels in BW5147 cells but interfered with the binding of NFATc2 to the IFN-γ and IL-2 promoters. These results indicate that kujigamberol inhibited IFN-γ and IL-2 mRNA expression by preventing the binding of NFATc2 to their promoters; therefore, it has potential as an immunosuppressive agent.

## 1. Introduction

T cells play a critical role in the adaptive immune system by responding and adapting to various biomechanical signals in order to regulate adhesion, migration, and trafficking and elicit immune functions [1]. T cells differentiate into distinct subsets that produce different sets of cytokines [2,3]. Cytokines are essential for the regulation of immune responses in a wide variety of target cells; however, excessive levels of cytokines cause inflammatory and allergic diseases [4,5].

Interleukin-2 (IL-2) is a primary cytokine that is predominantly produced by helper T cells, but it is also secreted by other cell types [6,7]. IL-2 has fundamental functions, such as supporting T-cell proliferation and survival and the differentiation of naïve T cells into effector and memory T cells [8,9]. IL-2 transcription is regulated by the proximal IL-2 promoter, which interacts with transcription factors, such as nuclear factor of activated T cell (NFAT), nuclear factor κB (NF-κB), and activated protein-1 (AP-1) [10,11]. Previous studies demonstrated that T-cell receptor stimulation induced increases in Ca^2+^ levels and activated protein kinases, which led to transcriptional activation mediated by NFAT, NF-κB, and AP-1 [12,13]. In addition to IL-2, T cells produce other cytokines, including interleukins, interferons, and members of the tumor necrosis factor superfamily [4,5].

Interferon-γ (IFN-γ) is a proinflammatory cytokine that plays a critical role in the regulation of immune responses, including antigen presentation and cytokine production [14,15]. It is mainly produced by T helper type 1 (Th1) cells, cytotoxic T cells, and natural killer (NK) cells [16,17]. NFAT and NF-κB were shown to be essential for IFN-γ transcription by interacting with the IFN-γ promoter [18]. In addition to these conventional transcription factors, lineage-specifying transcription factors are required to promote IFN-γ transcription through the epigenetic modification of the IFN-γ locus [18,19,20].

The NFAT family of transcription factors are regulated by Ca^2+^ signaling and are highly phosphorylated in the regulatory domain, which is a prerequisite for their sequestration in the cytosol [21,22]. Calcineurin, a serine/threonine phosphatase, is capable of directly dephosphorylating NFAT [21,22]. An increase in intracellular Ca^2+^ promotes the interaction of Ca^2+^-calmodulin with calcineurin and its phosphatase activity [23]. Upon dephosphorylation, NFAT is transcriptionally activated and translocates to the nucleus [24]. As a NFAT member, NFATc2 (also known as NFAT1) has been shown to play a critical role in the transcription of IFN-γ [25,26,27].

The T-box family member eomesodermin (Eomes) is regarded as a lineage-specifying transcription factor and consists of a T-box (DNA binding) domain and a transactivation domain [28,29]. Eomes plays an essential role in the differentiation and function of Th1 cells, cytotoxic T cells, and NK cells, including IFN-γ production [28,29]. Consistent with this notion, we reported that Eomes-transfected murine T-cell lymphomas acquired the ability to produce IFN-γ, even though their parental cell lines did not express detectable IFN-γ [30,31].

**Figure 1:**
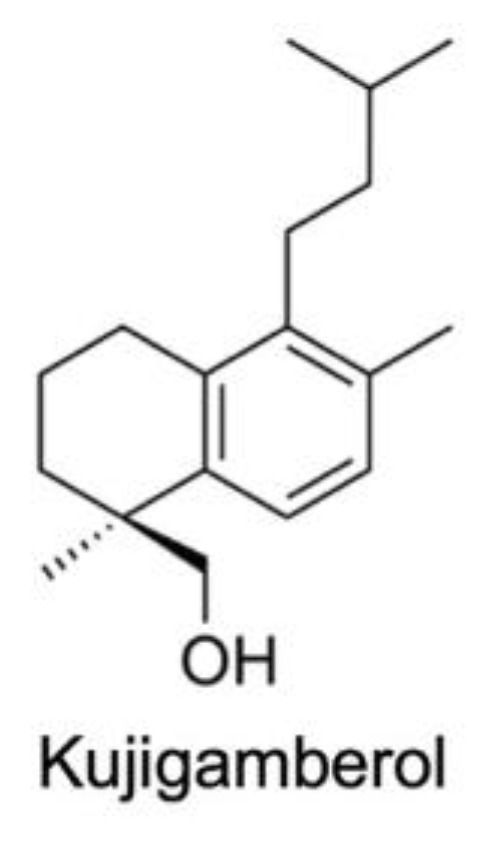
Structure of kujigamberol.

Kujigamberol (15,20-dinor-5,7,9-labdatrien-18-ol) (Figure 1) is a dinorlabdane compound that was originally identified in Kuji amber using screening based on mutant yeast hypersensitive to Ca^2+^-dependent signal transduction [32]. Previous studies demonstrated that kujigamberol exerted several biological effects, including anti-tumor activity against human leukemia HL-60 cells [32], anti-inflammatory activity in human vascular endothelial cells [33], and anti-allergic activity in rat basophilic leukemia RBL-2H3 cells and an in vivo rhinitis model using guinea pigs [34]. Our evaluation of the biological activity of kujigamberol in T cells showed that it suppressed IFN-γ and IL-2 expression in Eomes-transfected BW5147 cells. In the present study, we investigated the mechanisms by which kujigamberol inhibits T-cell cytokine expression.

## 2. Results

### 2.1. Kujigamberol Inhibited IFN-γ and IL-2 mRNA Expression in Murine T-Cell Lymphoma BW5147 Cells

Murine T-cell lymphoma BW5147 cells are capable of producing IL-2, but only a trace amount of IFN-γ, in response to phorbol 12-myristate 13-acetate (PMA) and A23187, which mimic T-cell receptor stimulation [35]. We previously showed that the T-box transcription factor Eomes enabled BW5147 cells to produce high levels of IFN-γ in response to PMA and ionomycin (IM) [30,31]. In the present study, we used Eomes-transfected BW5147 cells as a T-cell model to examine the effects of kujigamberol on cytokine production by T cells.

**Figure 2:**
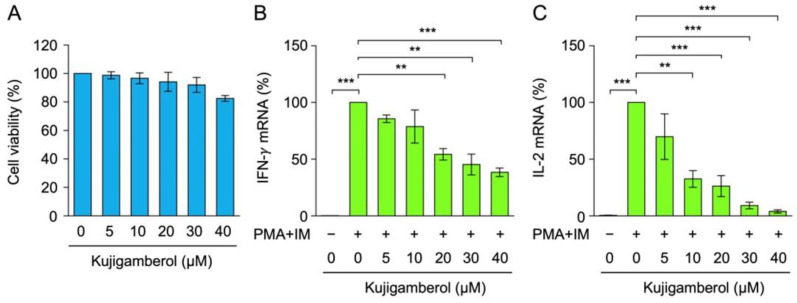
Kujigamberol inhibited IFN-γ and IL-2 mRNA expression in murine T-cell lymphoma BW5147 cells transfected with Eomes. (**A**) Eomes-transfected BW5147 cells were treated with (+) or without (−) serial dilutions of kujigamberol for 6 h at the indicated concentrations. Cell viability was evaluated by the MTT assay. Cell viability (%) is shown as the mean ± S.E. of three independent experiments. No significant differences were observed. (**B**,**C**) Eomes-transfected BW5147 cells were pretreated with (+) or without (−) serial dilutions of kujigamberol for 1 h and were then incubated with (+) or without (−) PMA (100 nM) plus IM (1 µM) for 6 h in the presence of kujigamberol at the indicated concentrations. IFN-γ and IL-2 mRNA expression was evaluated by RT-qPCR. IFN-γ mRNA (%) (**B**) and IL-2 mRNA (%) (**C**) are shown as the mean ± S.E. of three independent experiments. ** *p* < 0.01 and *** *p* < 0.001.

Eomes-transfected BW5147 cells were treated with kujigamberol for 6 h. Kujigamberol did not significantly affect cell viability at concentrations up to 40 µM (Figure 2A). Under these conditions, cytokine production at the mRNA level was assessed by reverse transcription-quantitative PCR (RT-qPCR). IFN-γ and IL-2 mRNA expression was up-regulated when Eomes-transfected BW5147 cells were stimulated with PMA and IM (Figure 2B,C). Kujigamberol decreased IFN-γ and IL-2 mRNA expression in a dose-dependent manner and at concentrations higher than 20 and 10 µM, respectively (Figure 2B,C). These results showed that kujigamberol inhibited IFN-γ and IL-2 mRNA up-regulated by PMA and IM.

### 2.2. Kujigamberol Inhibited IL-4 and Fas Ligand mRNA Expression in Eomes-Transfected BW5147 Cells

**Figure 3:**
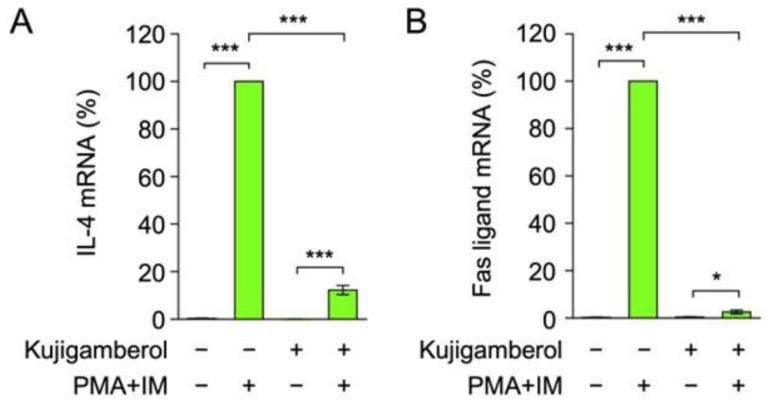
Kujigamberol inhibited IL-4 and Fas ligand mRNA expression in Eomes-transfected BW5147 cells. (**A**,**B**) Eomes-transfected BW5147 cells were pretreated with (+) or without (−) kujigamberol for 1 h, followed by stimulation with (+) or without (−) PMA (100 nM) plus IM (1 µM) for 6 h in the continued presence (+) or absence (−) of kujigamberol (40 µM). IL-4 and Fas ligand mRNA expression was evaluated by RT-qPCR. IL-4 mRNA (%) (**A**) and Fas ligand mRNA (%) (**B**) are shown as the mean ± S.E. of three independent experiments. * *p* < 0.05 and *** *p* < 0.001.

We further investigated the effects of kujigamberol on the production of other T-cell cytokines. The results obtained showed that IL-4 and Fas ligand mRNA expression was highly up-regulated in Eomes-transfected BW5147 cells stimulated with PMA and IM (Figure 3A,B). Kujigamberol markedly inhibited the up-regulation of IL-4 and Fas ligand mRNA expression (Figure 3A,B). Collectively, these results demonstrate that kujigamberol exerted potent inhibitory effects on cytokine mRNA expression in BW5147 cells.

### 2.3. Kujigamberol Inhibited IFN-γ mRNA Expression in the Murine Cytotoxic T-Cell Line CTLL-2

**Figure 4:**
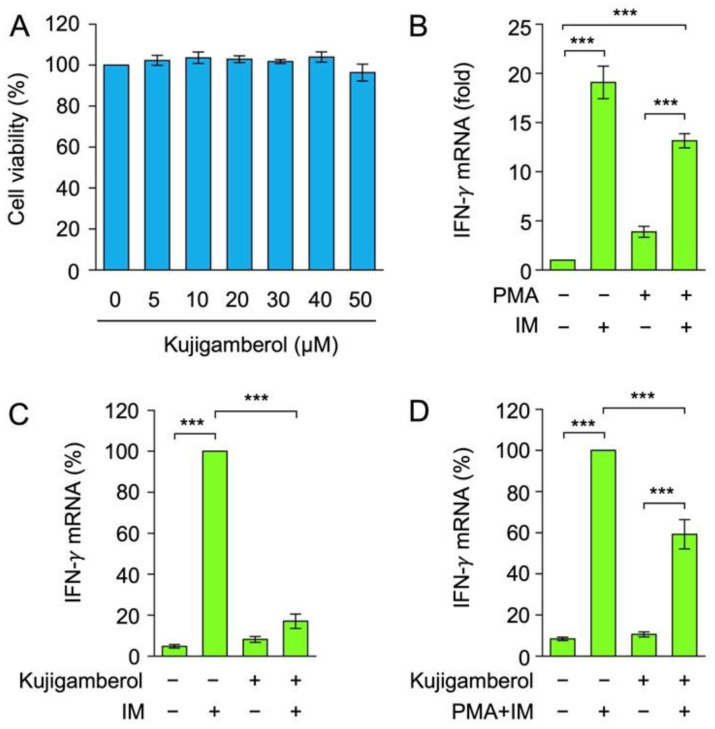
Kujigamberol inhibited IFN-γ mRNA expression in the murine cytotoxic T-cell line CTLL-2. (**A**) CTLL-2 cells were incubated with serial dilutions of kujigamberol for 6 h. Cell viability was evaluated by the MTT assay. Cell viability (%) is shown as the mean ± S.E. of three independent experiments. No significant differences were observed. (**B**–**D**) CTLL-2 cells were incubated with (+) or without (−) PMA (100 nM) and IM (1 µM) for 6 h (**B**). CTLL-2 cells were pretreated with (+) or without (−) kujigamberol for 1 h and were then incubated with (+) or without (−) IM (1 µM) (**C**) or PMA (100 nM) plus IM (1 µM) (**D**) for 6 h in the presence (+) or absence (−) of kujigamberol (50 µM). IFN-γ mRNA expression was evaluated by RT-qPCR. IFN-γ mRNA (fold) (**B**) and IFN-γ mRNA (%) (**C**,**D**) are shown as the mean ± S.E. of three independent experiments. *** *p* < 0.001.

In addition to helper T cells, IFN-γ is one of the cytokines produced by cytotoxic T cells and NK cells [16,17]. The murine cytotoxic T-cell line CTLL-2 has been reported to produce IFN-γ [36,37]. To investigate the effects of kujigamberol on cell viability, CTLL-2 cells were treated with serial dilutions of kujigamberol for 6 h. Kujigamberol did not affect CTLL-2 cell viability at concentrations up to 50 µM (Figure 4A). CTLL-2 cells were stimulated with PMA, IM, or both for 6 h. In contrast to BW5147 cells, IM alone was sufficient to up-regulate IFN-γ mRNA expression by approximately 20-fold (Figure 4B). Furthermore, the combination of PMA and IM up-regulated IFN-γ mRNA expression to a lesser extent than IM alone (Figure 4B). This may be due to the influence of PMA-induced AP-1 and NF-κB on IFN-γ transcription by competing with IM-induced NFAT as a major transcription factor in CTLL-2 cells. These results demonstrate that IM was the most suitable stimulation to promote IFN-γ expression in CTLL-2 cells.

CTLL-2 cells were treated with kujigamberol for 1 h and then stimulated with IM alone or the combination of PMA and IM. The pretreatment with kujigamberol suppressed IFN-γ mRNA expression by more than 80% in IM-stimulated CTLL-2 cells (Figure 4C). In contrast, kujigamberol inhibited IFN-γ mRNA expression by approximately 40% in PMA- and IM-stimulated CTLL-2 cells (Figure 4D), which was less than that in IM-stimulated CTLL-2 cells (Figure 4C). These results show that kujigamberol inhibited IFN-γ mRNA expression up-regulated by IM alone or PMA plus IM in CTLL-2 cells.

### 2.4. Kujigamberol Suppressed NFAT-Dependent Transcriptional Activity in Human Embryonic Kidney 293T Cells

**Figure 5:**
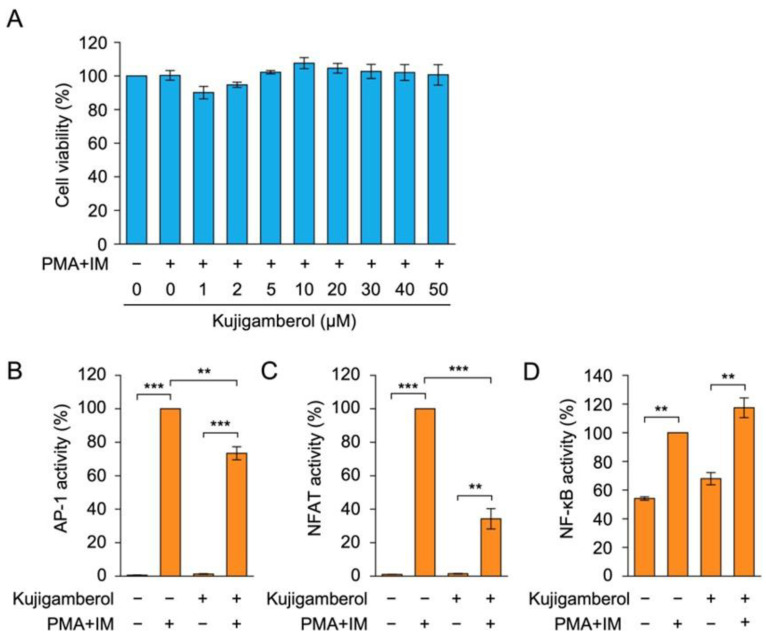
Kujigamberol inhibited NFAT-dependent luciferase reporter activity in human embryonic kidney 293T cells. (**A**) 293T cells were pretreated with (+) or without (−) serial dilutions of kujigamberol for 1 h and were then incubated with PMA (100 nM) plus IM (1 µM) for 6 h in the presence of kujigamberol at the indicated concentrations. Cell viability was evaluated by the MTT assay. Cell viability (%) is shown as the mean ± S.E. of three independent experiments. No significant differences were observed. (**B**–**D**) 293T cells were transfected with plasmid vectors encoding firefly luciferase reporters responsive to AP-1 (**B**), NFAT (**C**), and NF-κB (**D**), together with the *Renilla* luciferase reporter driven by the cytomegalovirus promoter. Transfected 293T cells were pretreated with (+) or without (−) kujigamberol (50 µM) for 1 h and were then treated with (+) or without (−) PMA (100 nM) and IM (1 µM) for 6 h. Firefly luciferase activity was normalized to *Renilla* luciferase activity. Luciferase activity (%) is shown as the mean ± S.E. of three independent experiments. ** *p* < 0.01 and *** *p* < 0.001.

We previously used human embryonic kidney 293T cells in a luciferase reporter assay on the IFN-γ promoter [30] because of their higher transfection efficiency for transient expression. To examine the effects of kujigamberol on cell viability, 293T cells were pretreated with kujigamberol for 1 h and then stimulated with PMA and IM for 6 h. The pretreatment with kujigamberol did not affect 293T cell viability at concentrations up to 50 µM (Figure 5A).

T-cell receptor stimulation or PMA and IM stimulation have been shown to activate transcription factors, such as AP-1, NF-κB, and NFAT, which then promote the transcription of cytokine genes, including IFN-γ and IL-2 [12,13]. To investigate the effects of kujigamberol on the transcription factors involved in cytokine production, 293T cells were transiently transfected with plasmid vectors encoding luciferase reporters responsive to AP-1, NFAT, and NF-κB. In transfected 293T cells, the PMA and IM stimulation up-regulated luciferase reporter activities for AP-1 and NFAT to high levels, but to a lesser extent for NF-κB (Figure 5B–D). Kujigamberol decreased AP-1-dependent luciferase activity by approximately 25%, whereas it inhibited NFAT-dependent luciferase activity by more than 65% (Figure 5B,C). In contrast, kujigamberol did not reduce NF-κB-dependent luciferase activity (Figure 5D). These results suggest that kujigamberol preferentially inhibited NFAT-dependent transcriptional activity over AP-1 or NF-κB.

### 2.5. Kujigamberol Did Not Affect NFATc2 Protein Expression in Eomes-Transfected BW5147 Cells

NFATs are dephosphorylated by the Ca^2+^- and calmodulin-dependent serine/threonine protein phosphatase calcineurin, leading to their activation and promotion of cytokine gene transcription [21,22]. The immunosuppressant FK506 (also known as tacrolimus) interacts with FK506-binding protein 12 (FKBP12), and the FK506–FKBP12 complex potently inhibits calcineurin activity and subsequent NFAT-dependent transcription [38]. We previously reported that FK506 inhibited the production of IFN-γ and IL-2 in Eomes-transfected BW5147 cells [30], indicating that NFAT plays an essential role in IFN-γ and IL-2 expression.

**Figure 6:**
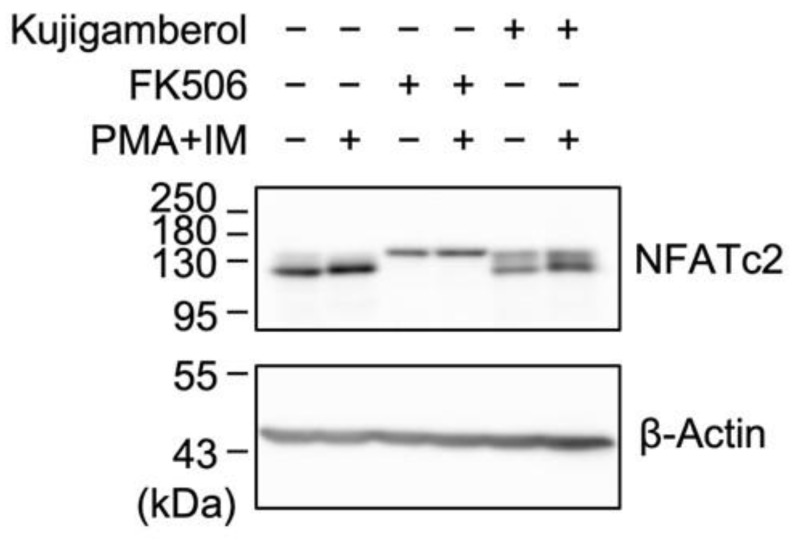
Kujigamberol did not markedly affect the NFATc2 protein in Eomes-transfected BW5147 cells. Eomes-transfected BW5147 cells were pretreated with (+) or without (−) kujigamberol or FK506 for 1 h and were then treated with (+) or without (−) PMA (100 nM) plus IM (1 µM) for 2 h in the presence of kujigamberol (40 µM) or FK506 (100 nM). Whole-cell lysates were prepared in at least two independent experiments. Blots were probed with anti-NFATc2 antibody and re-probed with β-actin antibody.

NFATc2 is one of five NFAT isoforms and is responsible for IFN-γ expression in T cells [25,26,27]. Eomes-transfected BW5147 cells have been shown to express the NFATc2 protein [31]. The luciferase reporter assay revealed that kujigamberol preferentially inhibited NFAT transcriptional activity (Figure 5C). Based on these results, we investigated the NFAT signaling pathway. Eomes-transfected BW5147 cells were pretreated with or without kujigamberol or FK506 for 1 h and then treated with PMA plus IM for 2 h. Whole-cell lysates were then prepared and analyzed via Western blotting. The mouse NFATc2 protein is composed of 927 amino acids and is estimated to be approximately 100 kDa. Consistent with this information, the NFATc2 protein was observed as a main band migrating near the 130 kDa marker (Figure 6). FK506 converted the NFATc2 protein to a band with a larger molecular weight (Figure 6), which may correspond to the phosphorylated form by preventing calcineurin-dependent dephosphorylation. The total amount of the NFATc2 protein was not affected by kujigamberol, whereas it was markedly decreased by FK506 (Figure 6). These results suggest that the mode of action of kujigamberol on the NFATc2 protein differed from that of FK506.

### 2.6. Kujigamberol Interfered with the Binding of NFATc2 to IFN-γ and IL-2 Promoters in Eomes-Transfected BW5147 Cells

**Figure 7:**
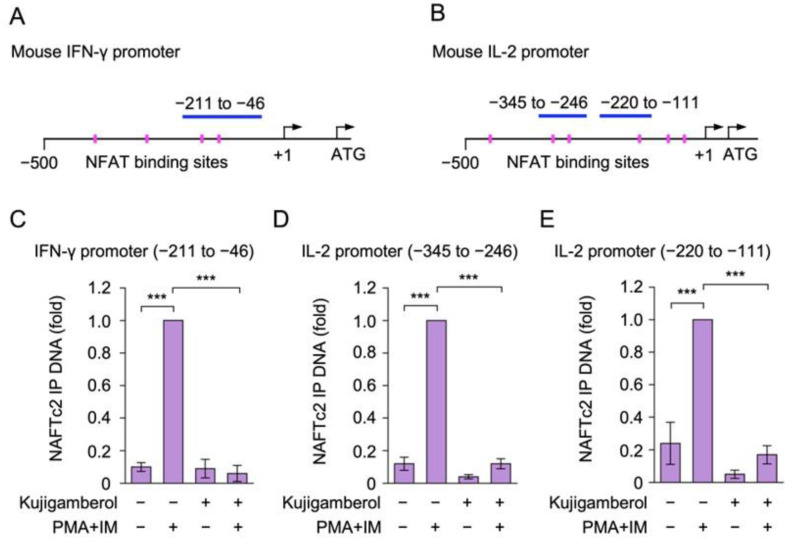
Kujigamberol reduced NFATc2 binding to IFN-γ and IL-2 promoters in Eomes-transfected BW5147 cells. (**A**,**B**) Consensus NFATc2 binding sites on the mouse IFN-γ promoter (**A**) and mouse IL-2 promoter (**B**) (magenta squares) are represented based on the JASPAR 2024 database. DNA regions amplified for the ChIP assay (blue lines) are shown. (**C**–**E**) Eomes-transfected BW5147 cells were pretreated with (+) or without (−) kujigamberol for 1 h and were then treated with (+) or without (−) PMA (100 nM) plus IM (1 µM) for 6 h in the presence (+) or absence (−) of kujigamberol (30 µM). The ChIP assay was performed using an anti-NFATc2 antibody. The amount of immunoprecipitated (IP) DNA for the IFN-γ promoter (−211 to −46) (**C**), IL-2 promoter (−345 to −246) (**D**), and IL-2 promoter (−220 to −111) (**E**) was measured by qPCR. NFATc2 IP DNA (fold) is shown as the mean ± S.E. of three independent experiments. *** *p* < 0.001.

We previously demonstrated that NFATc2 bound to the IFN-γ promoter in response to PMA and IM stimulation [31]. The JASPAR 2024 database [39] revealed that the IFN-γ promoter contains four consensus NFAT binding sites within −500 bp of the mouse IFN-γ promoter (Figure 7A and Figure S1), whereas six consensus NFAT binding sites exist within −500 bp of the IL-2 promoter (Figure 7B and Figure S2). Therefore, we investigated whether kujigamberol affected the binding of NFATc2 to the IFN-γ and IL-2 promoters using a chromatin immunoprecipitation (ChIP) assay. Eomes-transfected BW5147 cells were pretreated with kujigamberol and then stimulated with PMA and IM for 6 h. The PMA and IM stimulation markedly increased the binding of NFATc2 to the IFN-γ and IL-2 promoters (Figure 7C–E). The pretreatment with kujigamberol inhibited the binding of NFATc2 to the IFN-γ and IL-2 promoters in PMA- and IM-stimulated BW5147 cells (Figure 7C–E). These results show that kujigamberol inhibited the interaction of NFATc2 with the IFN-γ and IL-2 promoters.

## 3. Discussion

Kujigamberol is a major bioactive compound derived from Kuji amber [32]. It has been shown to exert anti-tumor effects on human leukemia HL-60 cells [32], anti-inflammatory activity in human vascular endothelial cells [33], and anti-allergic activity in rat basophilic leukemia RBL-2H3 cells and an in vivo rhinitis model [34]. In the present study, we revealed a novel biological effect of kujigamberol, namely the inhibition of cytokine expression by T cells. In Eomes-transfected BW5147 cells, kujigamberol inhibited the mRNA expression of IFN-γ, IL-2, IL-4, and the Fas ligand. IFN-γ mRNA expression was also suppressed by kujigamberol in CTLL-2 cells. Kujigamberol inhibited the binding of NFATc2 to the IFN-γ and IL-2 promoters in Eomes-transfected BW5147 cells. Previous studies reported that NFATc2 was indispensable for IFN-γ expression, while NFATc1 and NFATc2 were both required for IL-2 expression [25,26,27]. These findings show that kujigamberol inhibits IFN-γ transcription by preventing NFATc2 binding to the IFN-γ promoter.

IL-2 is a fundamental growth factor produced by T cells [8,9]. T cells differentiate into distinct subsets, including Th1 cells that produce IFN-γ and Th2 cells that secrete IL-4 [2,3]. Parental BW5147 cells are murine T-cell lymphoma cells that produce high levels of IL-2 and no or trace levels of IL-4 and IFN-γ, respectively [35]. Consistent with these findings, we previously demonstrated that BW5147 cells did not produce detectable levels of IFN-γ, whereas IFN-γ was clearly generated by Eomes-transfected BW5147 cells [30,31]. In contrast, BW5147 cells have been shown to express the Fas ligand in response to the calcium ionophore A23187 [40]. Eomes was previously found to up-regulate IFN-γ and Fas ligand expression in T cells [41,42]. It was previously shown that enforced expression of Eomes did not reduce IL-4 production in Th2 cells [43,44], whereas Eomes suppressed the IL-5 expression through inhibition of GATA3 in Th2 cells [44]. Our present results showed that Eomes-transfected BW5147 cells up-regulated the transcription of IFN-γ, IL-2, IL-4, and the Fas ligand in response to PMA and IM stimulation. However, it is currently unclear whether IL-4 expression is enhanced by Eomes in BW5147 cells or whether the present stimulation condition by PMA and IM is more potent in inducing IL-4 expression than that used in the previous study [35]. Using Eomes-transfected BW5147 cells as a T-cell model, we demonstrated that kujigamberol inhibited the mRNA expression of IFN-γ, IL-2, IL-4, and the Fas ligand as T-cell cytokines.

The results of the luciferase reporter assay showed that kujigamberol preferentially inhibited NFAT reporter activity over AP-1 reporter activity, whereas NF-κB reporter activity was unaffected. Previous studies reported that the binding sites for NFAT, NF-κB, and AP-1 were located in the IFN-γ promoter [45,46], IL-2 promoter [47,48], IL-4 promoter [49], and Fas ligand promoter [50]. Therefore, among the three types of transcription factors, NFAT appears to be the main target of kujigamberol. NFATc2 is one of five members of the NFAT family. Its role in the transcription of IFN-γ, IL-2, IL-4, and the Fas ligand has been demonstrated using gene knockout mice. Using NFATc2-deficient mice, NFATc2 was found to be indispensable for IFN-γ, IL-4, and Fas ligand expression in T cells, whereas IL-2 expression was not affected [25,26]. Furthermore, IL-2 expression was completely reduced in T cells deficient for NFATc1 and NFATc2 [51], suggesting that NFATc1 and NFATc2 have redundant roles in IL-2 expression. Consistent with these findings, FK506, which blocks the calcineurin–NFAT pathway, markedly inhibited the expression of IFN-γ and IL-2 in Eomes-transfected BW5147 cells [30]. Based on these findings, we focused on NFATc2 and investigated the effects of kujigamberol on NFATc2 protein and function.

The JASPAR 2024 database revealed the presence of four NFATc2 binding sites in the mouse IFN-γ promoter (−500 to −1) and six NFATc2 binding sites in the mouse IL-2 promoter (−500 to −1). We previously showed that PMA and IM stimulation increased the binding of NFATc2, the NF-κB subunit RelA, and Eomes to the IFN-γ promoter in Eomes-transfected BW5147 cells [30,31]. It has also been reported that T-cell receptor stimulation or PMA and IM stimulation up-regulated NFATc2 binding to the IL-2 promoter in T cells [52,53]. By using the same primers as those in ChIP assays in these studies, we demonstrated that kujigamberol inhibited the binding of NFATc2 to the IFN-γ and IL-2 promoters in Eomes-transfected BW5147 cells. However, unlike FK506, kujigamberol did not affect the total amount of the NFATc2 protein or its apparent molecular weight, suggesting that the phosphorylation status of the NFATc2 protein remained unchanged. Therefore, the mechanism by which kujigamerol inhibits NFATc2 binding activity differs from that of FK506, which inactivates calcineurin.

In our previous study, kujigamberol inhibited degranulation and Ca^2+^ mobilization in rat basophilic leukemia RBL-2H3 cells in response to A23187 [34]. The kujigamberol derivative 8-labden-15-oic acid also suppressed degranulation and Ca^2+^ mobilization in RBL-2H3 cells [54]. Kujigamberol did not inhibit calcineurin, even at 200 µM [32], whereas 8-labden-15-oic acid suppressed calcineurin at an IC_50_ value of 34.2 µM [54]. Collectively, these findings suggest that kujigamberol does not affect calcineurin but inhibits Ca^2+^-dependent cellular processes. Consistent with this notion, we demonstrated herein that the inhibitory mechanisms of kujigamberol and FK506 are different. In T cells, PMA mimics diacylglycerol and thereby activates protein kinases (e.g., protein kinase C and protein kinase θ), leading to the activation of the NF-κB and AP-1 pathways, whereas IM increases cytoplasmic Ca^2+^ through direct ionophore activity and the activation of calcium channels, which triggers the calcineurin–NFAT pathway [12]. Therefore, the Ca^2+^-dependent NFAT pathway appears to be more sensitive to kujigamberol than the protein-kinase-dependent NF-κB and AP-1 pathways. However, it is currently unclear whether kujigamberol reduced AP-1 reporter activity in 293T cells and inhibited IFN-γ mRNA expression in CTLL-2 cells induced by IM alone to a greater extent than the combination of PMA plus IM. Further experiments are needed to elucidate the molecular mechanisms by which kujigamberol inhibits cytokine production in T cells.

## 4. Materials and Methods

### 4.1. Cells

Murine T-cell lymphoma BW5147 cells (JCRB9002) were obtained from the National Institutes of Biomedical Innovation, Health and Nutrition JCRB Cell Bank (Osaka, Japan). BW5147 cells transfected with the pEF pGK puro expression vector encoding FLAG-Eomes (Eomes #2 BW5147 transfectant) were established and used in our previous studies [30,31]. The murine cytotoxic T-cell line CTLL-2 (RCB0637) and human embryonic kidney 293T cells (RCB2202) were obtained from the RIKEN BioResource Research Center (Tsukuba, Japan). Eomes-transfected BW5147 cells were maintained in RPMI 1640 medium (Thermo Fisher Scientific, Grand Island, NY, USA) supplemented with heat-inactivated fetal bovine serum (FBS) (Sigma-Aldrich, St. Louis, MO, USA) and penicillin–streptomycin mixed solution (stabilized) (Nacalai Tesque, Kyoto, Japan). CTLL-2 cells were maintained in RPMI 1640 medium supplemented with heat-inactivated FBS, penicillin–streptomycin mixed solution, and recombinant human IL-2 (100 U/mL) (PeproTech, Cranbury, NJ, USA). The 293T cells were maintained in DMEM medium supplemented with heat-inactivated FBS and penicillin–streptomycin mixed solution. All cells were incubated in a CO_2_ incubator at 37 °C and under 5% CO_2_ conditions.

### 4.2. Reagents

Kujigamberol was prepared as previously described [32]. FK506 (Cayman Chemical, Ann Arbor, MI, USA), IM (Merck Millipore, Darmstadt, Germany), and PMA (Wako Pure Chemical Industries, Osaka, Japan) were commercially purchased.

### 4.3. Antibodies

Primary antibodies reactive to NFATc2 (4G6-G5; Santa Cruz Biotechnology, Dallas, TX, USA) and β-actin (AC-15; Sigma-Aldrich) and a peroxidase-conjugated anti-mouse IgG (H+L) antibody (115-035-146; Jackson Immuno Research Laboratories, West Grove, PA, USA) were used. An anti-NFATc2 antibody (4G6-G5) was used for both Western blotting and immunoprecipitation.

### 4.4. Plasmid Vectors

Plasmid vectors encoding firefly luciferase reporters responsive to AP-1, NF-κB, and NFAT (kindly provided by Prof. Ralph C. Budd) were used as previously described [55]. A plasmid vector encoding a cytomegalovirus promoter-driven *Renilla* luciferase reporter was employed as previously described [56].

### 4.5. Cell Viability Assay

Cells (5 × 10^4^ cells/well for BW5147 cells, 5 × 10^4^ cells/well for CTLL-2 cells, and 2 × 10^4^ cells/well for 293T cells) were seeded on 96-well plates and preincubated overnight. Cell viability was assessed by the formazan formation of 3-(4,5-dimethyl-2-thiazolyl)-2,5-diphenyltetrazolium bromide (MTT). Cells were treated with reagents and then incubated with MTT (500 µg/mL) for the last 2 h. MTT formazan was solubilized with sodium dodecyl sulfate overnight at the final concentration of 5%. Absorbance at 570 nm was measured using an iMark microplate reader (Bio-Rad Laboratories, Hercules, CA, USA).

### 4.6. RT-qPCR

Cells (6 × 10^5^ cells/dish for BW5147 cells and 5 × 10^5^ cells/dish for CTLL-2 cells) were seeded on 35 mm dishes and preincubated overnight. Cells were treated with reagents, harvested by centrifugation (800× *g*, 5 min), and treated with Sepasol RNA I Super G (Nacalai Tesque) according to the manufacturer’s protocol. Total RNA was purified and solubilized with DEPC water (Nacalai Tesque). Total RNA was converted into cDNA using ReverTra Ace^®^ (TOYOBO, Osaka, Japan) and oligo (dT)_20_. mRNA levels were measured by the Thermal Cycler Dice^®^ Real Time System Lite (Takara Bio, Kusatsu, Japan) using primer pairs [57,58,59] (Table S1). Primer-specific standard curves were used for the quantification of initial DNA. The PCR conditions were 94 °C for 3 min, followed by 45 cycles at 95 °C for 20 s, 58 °C for 30 s, and 72 °C for 30 s.

### 4.7. Reporter Assay

The 293T cells (5 × 10^4^ cells/well) were seeded on 24-well plates and preincubated overnight. Cells were transfected with plasmid vectors encoding firefly luciferase reporters responsive to AP-1, NF-κB, and NFAT, together with a plasmid vector encoding a cytomegalovirus promoter-driven *Renilla* luciferase reporter using the calcium phosphate method. Transfected cells were treated with reagents, harvested by centrifugation (800× *g*, 5 min), and washed with phosphate-buffered saline (PBS). Cells were lysed with digitonin lysis buffer consisting of 1% Triton X-100, 50 mM Tris-HCl (pH 7.4), 2 mM dithiothreitol, 2 mM sodium vanadate, and the cOmplete^TM^ Protease Inhibitor Cocktail (Sigma-Aldrich) and incubated on ice for 15 min, followed by centrifugation (15,300× *g*, 5 min) to collect the supernatants. Luciferase activities were measured using Lumitester C-110 (Kikkoman Biochemifa, Tokyo, Japan). Firefly luciferase activities were normalized to *Renilla* luciferase activities.

### 4.8. Western Blotting

The preparation of whole-cell lysates and Western blotting were performed as previously described [30]. In brief, BW5147 cells (6 × 10^5^ cells/dish) were seeded on 35 mm dishes and preincubated overnight. Cells were treated with reagents, harvested by centrifugation (800× *g*, 5 min), and washed with PBS. Cells were then lysed using Triton X-100 lysis buffer consisting of 50 mM Tris-HCl (pH 7.4), 1% Triton X-100, 2 mM dithiothreitol, 2 mM sodium orthovanadate, and the protease inhibitor cocktail cOmplete^TM^ (Sigma-Aldrich) and treated via sonication to prepare whole-cell lysates. The anti-NFATc2 antibody (4G6-G5) was used for Western blotting. Blots were visualized and captured using Amersham Imager 680 (GE Healthcare Japan, Tokyo, Japan). Membranes were treated with stripping solution (Fujifilm Wako Pure Chemical Corporation, Richmond, VA, USA) and were then subjected to additional Western blotting using the anti-β-actin antibody (AC-15).

### 4.9. ChIP Assay

BW5147 cells (5 × 10^6^ cells/dish) were seeded on 100 mm dishes and preincubated overnight. Cells were treated with reagents and fixed via the direct addition of formaldehyde to the culture medium. The ChIP assay was performed as previously described [30]. Immunoprecipitation was conducted with the anti-NFATc2 antibody (4G6-G5) (2 µg). Control IgG was not used in this study because the specificity of the anti-NFATc2 antibody (mouse IgG2a) used for the ChIP assay was validated in our previous study using mouse IgG2a (MOPC-173) as a negative control [60]. Immunoprecipitated DNA and input DNA were analyzed by qPCR using primers for the mouse IFN-γ promoter and mouse IL-2 promoter [52,53,61] (Table S2).

### 4.10. Statistical Analysis

Experiments were repeated to confirm reproducibility. Regarding quantification, means ± standard errors were evaluated based on at least three independent experiments. Each figure caption indicates the number of experiments. One-way ANOVA followed by Tukey’s test were used to assess the significance of the differences (KaleidaGraph software version 4.5.1; Hulinks, Tokyo, Japan). *p*-values less than 0.05 were considered to be significant.

## 5. Conclusions

The present study demonstrated that kujigamberol reduced the mRNA expression of IFN-γ, IL-2, IL-4, and the Fas ligand in T cells. Kujigamberol preferentially suppressed the transcriptional activity of NFAT and interfered with the DNA binding activity of NFATc2. The NFAT signaling pathway is activated by Ca^2+^- and calmodulin-dependent calcineurin [21,22]. The calcineurin inhibitor FK506 has been used clinically as an immunosuppressant in organ transplantation [62,63]. In a mechanistic manner distinct from FK506, the present study showed that kujigamberol inhibited IFN-γ and IL-2 mRNA expression by preventing NFATc2 binding to their promoters. Therefore, kujigamberol is expected to be a potential lead for the development of immunosuppressive agents.

## Data Availability

Data will be made available upon reasonable request.

## Supplementary Materials

The following supporting information can be downloaded at: https://www.mdpi.com/article/10.3390/molecules30102214/s1, Figure S1: NFATc2 binding sites of the mouse IFN-γ promoter; Figure S2: NFATc2 binding sites of the mouse IL-2 promoter; Figure S3: Original blots in Figure 6; Table S1: Primers used for RT-qPCR; Table S2: Primers used for the ChIP assay.

## References

[1] Chen W., Zhu C. (2013). Mechanical regulation of T-cell functions. Immunol. Rev..

[2] Raphael I., Nalawade S., Eagar T.N., Forsthuber T.G. (2015). T cell subsets and their signature cytokines in autoimmune and inflammatory diseases. Cytokine.

[3] Dong C. (2021). Cytokine Regulation and Function in T Cells. Annu. Rev. Immunol..

[4] Turner M.D., Nedjai B., Hurst T., Pennington D.J. (2014). Cytokines and chemokines: At the crossroads of cell signalling and inflammatory disease. Biochim. Biophys. Acta..

[5] Liu C., Chu D., Kalantar-Zadeh K., George J., Young H.A., Liu G. (2021). Cytokines: From clinical significance to quantification. Adv. Sci..

[6] Rosenberg S.A. (2014). IL-2: The first effective immunotherapy for human cancer. J. Immunol..

[7] Damoiseaux J. (2020). The IL-2—IL-2 receptor pathway in health and disease: The role of the soluble IL-2 receptor. Clin. Immunol..

[8] Liao W., Lin J.X., Leonard W.J. (2011). IL-2 family cytokines: New insights into the complex roles of IL-2 as a broad regulator of T helper cell differentiation. Curr. Opin. Immunol..

[9] Shouse A.N., LaPorte K.M., Malek T.R. (2024). Interleukin-2 signaling in the regulation of T cell biology in autoimmunity and cancer. Immunity.

[10] Bunting K., Wang J., Shannon M.F. (2006). Control of interleukin-2 gene transcription: A paradigm for inducible, tissue-specific gene expression. Vitam. Horm..

[11] Crispín J.C., Tsokos G.C. (2009). Transcriptional regulation of IL-2 in health and autoimmunity. Autoimmun. Rev..

[12] Shah K., Al-Haidari A., Sun J., Kazi J.U. (2021). T cell receptor (TCR) signaling in health and disease. Signal Transduct. Target Ther..

[13] Hermann-Kleiter N., Baier G. (2010). NFAT pulls the strings during CD4^+^ T helper cell effector functions. Blood.

[14] Schroder K., Hertzog P.J., Ravasi T., Hume D.A. (2004). Interferon-γ: An overview of signals, mechanisms and functions. J. Leukoc. Biol..

[15] Jorgovanovic D., Song M., Wang L., Zhang Y. (2020). Roles of IFN-γ in tumor progression and regression: A review. Biomark. Res..

[16] Rožman P., Švajger U. (2018). The tolerogenic role of IFN-γ. Cytokine Growth Factor Rev..

[17] Burke J.D., Young H.A. (2019). IFN-γ: A cytokine at the right time, is in the right place. Semin. Immunol..

[18] Balasubramani A., Mukasa R., Hatton R.D., Weaver C.T. (2010). Regulation of the *Ifng* locus in the context of T-lineage specification and plasticity. Immunol. Rev..

[19] Wilson C.B., Rowell E., Sekimata M. (2009). Epigenetic control of T-helper-cell differentiation. Nat. Rev. Immunol..

[20] Aune T.M., Collins P.L., Collier S.P., Henderson M.A., Chang S. (2013). Epigenetic activation and silencing of the gene that encodes IFN-γ. Front. Immunol..

[21] Li H., Rao A., Hogan P.G. (2011). Interaction of calcineurin with substrates and targeting proteins. Trends Cell Biol..

[22] Qin J.J., Nag S., Wang W., Zhou J., Zhang W.D., Wang H., Zhang R. (2014). NFAT as cancer target: Mission possible?. Biochim. Biophys. Acta..

[23] Dewenter M., von der Lieth A., Katus H.A., Backs J. (2017). Calcium signaling and transcriptional regulation in cardiomyocytes. Circ. Res..

[24] Fric J., Zelante T., Wong A.Y.W., Mertes A., Yu H.B., Ricciardi-Castagnoli P. (2012). NFAT control of innate immunity. Blood.

[25] Hodge M.R., Ranger A.M., Charles de la Brousse F., Hoey T., Grusby M.J., Glimcher L.H. (1996). Hyperproliferation and dysregulation of IL-4 expression in NF-ATp-deficient mice. Immunity.

[26] Kiani A., García-Cózar F.J., Habermann I., Laforsch S., Aebischer T., Ehninger G., Rao A. (2001). Regulation of interferon-γ gene expression by nuclear factor of activated T cells. Blood.

[27] Teixeira L.K., Fonseca B.P.F., Vieira-de-Abreu A., Barboza B.A., Robbs B.K., Bozza P.T., Viola J.P. (2005). IFN-γ production by CD8^+^ T cells depends on NFAT1 transcription factor and regulates Th differentiation. J. Immunol..

[28] Zhang J., Marotel M., Fauteux-Daniel S., Mathieu A.L., Viel S., Marçais A., Walzer T. (2018). T-bet and Eomes govern differentiation and function of mouse and human NK cells and ILC1. Eur. J. Immunol..

[29] Pritchard G.H., Kedl R.M., Hunter C.A. (2019). The evolving role of T-bet in resistance to infection. Nat. Rev. Immunol..

[30] Fukuoka N., Harada M., Nishida A., Ito Y., Shiota H., Kataoka T. (2016). Eomesodermin promotes interferon-γ expression and binds to multiple conserved noncoding sequences across the *Ifng* locus in mouse thymoma cell lines. Genes Cells.

[31] Harada M., Vo N.T., Nakao A., Tanigaki R., Fukuoka N., Nishida A., Kataoka T. (2020). Eomesodermin promotes interaction of RelA and NFATc2 with the *Ifng* promoter and multiple conserved noncoding sequences across the *Ifng* locus in mouse lymphoma BW5147 cells. Immunol. Lett..

[32] Kimura K., Minamikawa Y., Ogasawara Y., Yoshida J., Saitoh K., Shinden H., Ye Y.Q., Takahashi S., Miyakawa T., Koshino H. (2012). Kujigamberol, a new dinorlabdane diterpenoid isolated from 85 million years old Kuji amber using a biotechnological assay. Fitoterapia.

[33] Fukuhara S., Tanigaki R., Kimura K., Kataoka T. (2018). Kujigamberol interferes with pro-inflammatory cytokine-induced expression of and N-glycan modifications to cell adhesion molecules at different stages in human umbilical vein endothelial cells. Int. Immunopharmacol..

[34] Maruyama M., Kobayashi M., Uchida T., Shimizu E., Higashio H., Ohno M., Uesugi S., Kimura K. (2018). Anti-allergy activities of Kuji amber extract and kujigamberol. Fitoterapia.

[35] Hagiwara H., Yokota T., Luh J., Lee F., Arai K., Arai N., Zlotnik A. (1988). The AKR thymoma BW5147 is able to produce lymphokines when stimulated with calcium ionophore and phorbol ester. J. Immunol..

[36] Li Y.Q., Kobayashi M., Yuan L., Wang J., Matsushita K., Hamada J., Kimura K., Yagita H., Okumura K., Hosokawa M. (1998). Protein kinase C mediates the signal for interferon-γ mRNA expression in cytotoxic T cells after their adhesion to laminin. Immunology.

[37] Lv S., Yi P.F., Shen H.Q., Zhang L.Y., Dong H.B., Wu S.C., Xia F., Guo X., Wei X.B., Fu B.D. (2014). Ginsenoside Rh2-B1 stimulates cell proliferation and IFN-γ production by activating the p38 MAPK and ERK-dependent signaling pathways in CTLL-2 cells. Immunopharmacol. Immunotoxicol..

[38] Sieber M., Baumgrass R. (2009). Novel inhibitors of the calcineurin/NFATc hub–Alternatives to CsA and FK506?. Cell Commun. Signal..

[39] Rauluseviciute I., Riudavets-Puig R., Blanc-Mathieu R., Castro-Mondragon J.A., Ferenc K., Kumar V., Lemma R.B., Lucas J., Chèneby J., Baranasic D. (2024). JASPAR 2024: 20th anniversary of the open-access database of transcription factor binding profiles. Nucleic Acids Res..

[40] Benbijja M., Mellouk A., Bobé P. (2014). Sensitivity of leukemic T-cell lines to arsenic trioxide cytotoxicity is dependent on the induction of phosphatase B220/CD45R expression at the cell surface. Mol. Cancer..

[41] Pearce E.L., Mullen A.C., Martins G.A., Krawczyk C.M., Hutchins A.S., Zediak V.P., Banica M., DiCioccio C.B., Gross D.A., Mao C.A. (2003). Control of effector CD8^+^ T cell function by the transcription factor Eomesodermin. Science.

[42] Eshima K., Chiba S., Suzuki H., Kokubo K., Kobayashi H., Iizuka M., Iwabuchi K., Shinohara N. (2012). Ectopic expression of a T-box transcription factor, eomesodermin, renders CD4^+^ Th cells cytotoxic by activating both perforin- and FasL-pathways. Immunol. Lett..

[43] Yagi R., Junttila I.S., Wei G., Urban J.F., Zhao K., Paul W.E., Zhu J. (2010). The transcription factor GATA3 actively represses RUNX3 protein-regulated production of interferon-γ. Immunity.

[44] Endo Y., Iwamura C., Kuwahara M., Suzuki A., Sugaya K., Tumes D.J., Tokoyoda K., Hosokawa H., Yamashita M., Nakayama T. (2011). Eomesodermin controls interleukin-5 production in memory T helper 2 cells through inhibition of activity of the transcription factor GATA3. Immunity.

[45] Aune T.M., Penix L.A., Rincón M.R., Flavell R.A. (1997). Differential transcription directed by discrete gamma interferon promoter elements in naive and memory (effector) CD4 T cells and CD8 T cells. Mol. Cell. Biol..

[46] Sica A., Dorman L., Viggiano V., Cippitelli M., Ghosh P., Rice N., Young H.A. (1997). Interaction of NF-κB and NFAT with the interferon-γ promoter. J. Biol. Chem..

[47] Rooney J.W., Sun Y.L., Glimcher L.H., Hoey T. (1995). Novel NFAT sites that mediate activation of the interleukin-2 promoter in response to T-cell receptor stimulation. Mol. Cell. Biol..

[48] Walters R.D., Drullinger L.F., Kugel J.F., Goodrich J.A. (2013). NFATc2 recruits cJun homodimers to an NFAT site to synergistically activate interleukin-2 transcription. Mol. Immunol..

[49] Li-Weber M., Giaisi M., Baumann S., Pálfi K., Krammer P.H. (2004). NF-κB synergizes with NF-AT and NF-IL6 in activation of the IL-4 gene in T cells. Eur. J. Immunol..

[50] Kavurma M.M., Khachigian L.M. (2003). Signaling and transcriptional control of Fas ligand gene expression. Cell Death Differ..

[51] Peng S.L., Gerth A.J., Ranger A.M., Glimcher L.H. (2001). NFATc1 and NFATc2 together control both T and B cell activation and differentiation. Immunity.

[52] Wu Y., Borde M., Heissmeyer V., Feuerer M., Lapan A.D., Stroud J.C., Bates D.L., Guo L., Han A., Ziegler S.F. (2006). FOXP3 controls regulatory T cell function through cooperation with NFAT. Cell.

[53] Ishihara S., Schwartz R.H. (2011). Two-step binding of transcription factors causes sequential chromatin structural changes at the activated IL-2 promoter. J. Immunol..

[54] Abe T., Kobayashi M., Okawa Y., Inui T., Yoshida J., Higashio H., Shinden H., Uesugi S., Koshino H., Kimura K. (2016). Yeast Ca^2+^-signal transduction inhibitors isolated from Dominican amber prevent the degranulation of RBL-2H3 cells through the inhibition of Ca^2+^-influx. Fitoterapia.

[55] Dohrman A., Kataoka T., Cuenin S., Russell J.Q., Tschopp J., Budd R.C. (2005). Cellular FLIP (long form) regulates CD8^+^ T cell activation through caspase-8-dependent NF-κB activation. J. Immunol..

[56] Matsuda I., Matsuo K., Matsushita Y., Haruna Y., Niwa M., Kataoka T. (2014). The C-terminal domain of the long form of cellular FLICE-inhibitory protein (c-FLIP_L_) inhibits the interaction of the caspase 8 prodomain with the receptor-interacting protein 1 (RIP1) death domain and regulates caspase 8-dependent nuclear factor κB (NF-κB) activation. J. Biol. Chem..

[57] Casteels K.M., Mathieu C., Waer M., Valckx D., Overbergh L., Laureys J.M., Bouillon R. (1998). Prevention of type I diabetes in nonobese diabetic mice by late intervention with nonhypercalcemic analogs of 1,25-dihydroxyvitamin D_3_ in combination with a short induction course of cyclosporin A. Endocrinology.

[58] Orsatti C.L., Missima F., Pagliarone A.C., Sforcin J.M. (2010). Th1/Th2 cytokines’ expression and production by propolis-treated mice. J. Ethnopharmacol..

[59] Pinhu L., Qin Y., Xiong B., You Y., Li J., Sooranna S.R. (2014). Overexpression of Fas and FasL is associated with infectious complications and severity of experimental severe acute pancreatitis by promoting apoptosis of lymphocytes. Inflammation.

[60] Tanaka Y., Nakao A., Miyake Y., Higashi Y., Tanigaki R., Kataoka T. (2021). Small molecule inhibitors targeting nuclear factor κB activation markedly reduce expression of interleukin-2, but not interferon-γ, induced by phorbol esters and calcium ionophores. Int. J. Mol. Sci..

[61] Chang S., Aune T.M. (2007). Dynamic changes in histone-methylation “marks” across the locus encoding interferon-γ during the differentiation of T helper type 2 cells. Nat. Immunol..

[62] Dheer D., Jyoti, Gupta P.N., Shankar R. (2018). Tacrolimus: An updated review on delivering strategies for multifarious diseases. Eur. J. Pharm. Sci..

[63] Ong S.C., Gaston R.S. (2021). Thirty years of tacrolimus in clinical practice. Transplantation.

