# Supplementary material for: Kujigamberol Inhibits IFN-γ and IL-2 mRNA Expression and NFATc2 Binding to Their Promoters in Response to a Phorbol Ester and Ionomycin Stimulation
Source: Molecules. 2025 May 19;30(10):2214. doi: 10.3390/molecules30102214 (PMC12114272; doi:10.3390/molecules30102214)
Supplement: Supplementary file 1 [file molecules-30-02214-s001.zip › molecules-3629901-supplementary.pdf]

Mouse IFN-γ promoter

−500

TCCTTGCTTTTCTGGTCATTTGCAAGAAAAGTTTGAAAAGGCTTCCCCCATTGCATGGTT

TGAGAAGCCCAAGAGTTTCCTCATGGTTTGAGAAGCCCAAGAG**TTTCCTTT**TATTCAGCC

GTCCCCAACCACAAACAAAGGCTCCCTGTGCTGTGCTCTGTGGATGAGAAATTCACATTA

CAAGGGCAAAAAGGGGGAGACGTAAAAGCAA**TTTCCAGC**CCCCACCCCAAATGGTGTGAA

−211

GTAAAAGTGCTTTCAGAGAATCCCACAAGAATGGCACAGGTGGGCACAG**CGGGGCTGTCT**

CATCGTCAGAGAGCCCAAGGAGTCG**AAAGGAAA**CTCTAACATGCCACAAAACCATAGCTG

T**AATGCAAA**GTAACTTAGCTCCCCCCACCTATCTGTCACCATCTTAAAAAAAAAAAAAAA

−46

CCAAAAAAAAAC**TTGTGAAAATACGTAATCCCGAG**GAGCCTTCGATCAGGTATAAAACTG

+1

GAAGCCAGAGAGGTGCAGGCTATAGCTGCCATCGGCTGACCTAGAGAAGACACATCAGCT

GATCCTTTGGACCCTCTGACTTGAGACAGAAGTTCTGGGCTTCTCCTCCTGCGGCCTAGC

Translation

TCTGAGACAATGAACGCTAC

Figure S1. NFATc2 binding sites of the mouse IFN-γ promoter. The transcription start site (+1) and translation start site are indicated. Primers used for the ChIP assay (red) are shown. Consensus NFATc2 binding sites (arrows) in the mouse IFN-γ promoter are represented based on the JASPAR 2024 database.

Mouse IL-2 promoter

−500

CTGTGTGCAATTAGCTCATTGTGTAGATAAGAAGGTAAAACCATCTTGAA**ACAGGAAA**CC

AATATCCTTCCTGTCTAATCAACAAATCTAAAAGATTTATTCTTTTCATCTATCTCCTCT

−345

TGCGTTTGTCCACCACAACAGGCTGCTTACAGGTT**CAGGATGGTTTTGACAAAGAGAACA**

TTTTCATGAGTTACTTTTGTGTCTCCACCCCAA**AGAGGAAA**ATTTGTTT**CATACAGAAGG**

−246 −220

CGTTCATTGTATGAATTAAAACTGCCACCTAAGTGTGGGC**TAACCCGACCAAGAGGGATT**

TCACCTAAATCCATTCAGTCAGTGTATGGGGGTTTAAAGAAATTCCAGAGAGTCATCAGA

−111

AGAGGAAAAA**CAAAGGTAATGCTTTCTGCC**ACACAGGTAGACTCTTTGAAAATATGTGTA

A**TATGTAAA**ACATCGTGACACCCCCATATTATT**TTTCCAGC**ATTAACAGTATAAATTGCC

+1

TCCCATGCTGAAGAGCTGCCTATCACCCTTGCTAATCACTCCTCACAGTGACCTCAAGTC

Translation

CTGCAGGCATGTACAGCATGCAG

Figure S2. NFATc2 binding sites of the mouse IL-2 promoter. The transcription start site (+1) and translation start site are indicated. Primers used for the ChIP assay (red and blue) are shown. Consensus NFATc2 binding sites (arrows) in the mouse IL-2 promoter are represented based on the JASPAR 2024 database.

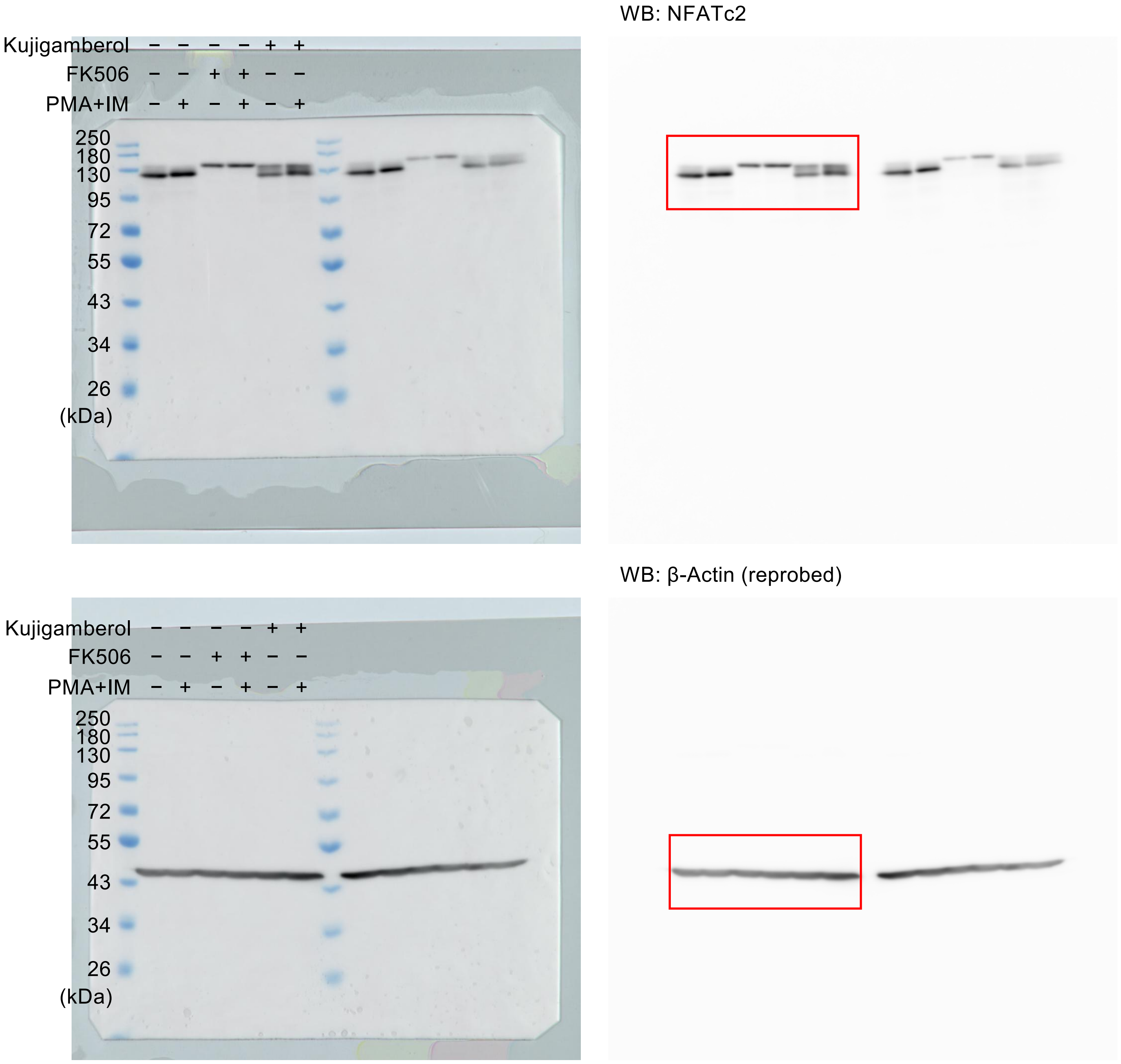

Figure S3. Original blots in Figure 6

Table S1. Primers used for RT-qPCR

Gene	Direction	Sequence	Size (bp)	Ref.
IFN-γ (mouse)	Forward<br>Reverse	5′-AGCCAAGACTGTGATTGCGG-3′<br>5′-TGCTGTCTGGCCTGCTGTTAA-3′	196	57
IL-2 (mouse)	Forward<br>Reverse	5′-CCTGAGCAGGATGGAGAATTACA-3′<br>5′-TCCAGAACATGCCGCAGAG-3′	141	57
IL-4 (mouse)	Forward<br>Reverse	5′-AGATGGATGTGCCAAACGTCCTCA-3′<br>5′-AATATGCGAAGCACCTTGGAAGCC-3′	88	58
FasL (mouse)	Forward<br>Reverse	5′-GCAGAAGGAACTGGCAGAAC-3′<br>5′-TTAAATGGGCCACACTCCTC-3′	128	59
β-Actin (mouse)	Forward<br>Reverse	5′-AGAGGGAAATCGTGCGTGAC-3′<br>5′-CAATAGTGATGACCTGGCCGT-3′	138	57

Table S2. Primers used for the ChIP assay

Promoter region	Direction	Sequence	Ref.
IFN-γ (mouse)<br>−211 to −46	Forward<br>Reverse	5′-CGGGGCTGTCTCATCGTC-3′<br>5′-CTCGGGATTACGTATTTTCACAA-3′	61
IL-2 (mouse)<br>−345 to −246	Forward<br>Reverse	5′-CAGGATGGTTTTGACAAAGAGAACA-3′<br>5′-TTCATACAATGAACGCCTTCTGTATG-3′	52
IL-2 (mouse)<br>−220 to −111	Forward<br>Reverse	5′-TAACCCGACCAAGAGGGATT-3′<br>5′-GGCAGAAAGCATTACCTTTG-3′	53

Locations relative to transcription start sites are based on the NCBI database: mouse IFN-γ (Gene ID: 15978) and mouse IL-2 (Gene ID: 16183).
